# A Novel CRYBB2 Silent Variant in Autosomal Dominant Congenital Cataracts (ADCC) in Pakistani families

**DOI:** 10.12669/pjms.39.5.7061

**Published:** 2023

**Authors:** Maryam Hussain, Khushi Muhammad, Muhammad Khan, Aziz Ud Din

**Affiliations:** 1 Maryam Hussain, M.Phil. Department of Biotechnology and Genetic Engineering, Hazara University Mansehra, 21120, Khyber Pakhtunkhwa, Pakistan; 2 Khushi Muhammad, PhD. Associate Professor, Department of Life Science, Imperial College London, Sir Alex Fleming Building South, Kensington Campus London, SW7 2AZ, United Kingdom; 3 Muhammad Khan, PhD. Assistant Professor, Department of Biotechnology and Genetic Engineering, Hazara University Mansehra, 21120, Khyber Pakhtunkhwa, Pakistan; 4 Aziz Ud Din, PhD. Assistant Professor, Department of Biotechnology and Genetic Engineering, Hazara University Mansehra, 21120, Khyber Pakhtunkhwa, Pakistan

**Keywords:** Congenital cataract, *β*-crystalline, lamellar cataracts Nuclear cataract, Red reflex examination and Swiss-model

## Abstract

**Objective:**

Congenital Cataract is a type of ophthalmic genetic disorder that appears at birth or in early childhood. Among 30 genes, *CRYBB2* is one of the most common and a water-soluble protein of lens’s that code for the βB2-crystallin. This study aimed to investigate the novel silent mutation in *CRYBB2* of exon six in the Pakistani families of Autosomal Dominant Congenital Cataracts (ADCC).

**Methods:**

It is a family-based study that presents three to five-generations of two Pakistani families. Data and blood samples from the families were collected from January to August 2019 from LRBT (Layton Rahmatullah Benevolent Trust) Hospital, Mansehra, Pakistan. We only included patients >15 years old. Before enrollment in the current study, each patient obtained a thorough optical examination. Samples were moved to the molecular lab using the collection and storage method. The phenol-chloroform technique was used to extract the DNA. The technique of Sanger sequencing was used to find any potential mutation in some of the selected families. Statistical and bioinformatics analysis were carried out.

**Results:**

By using bioinformatics tools, the novel silent mutation was identified. Heterozygous silent mutation of *CRYBB2* of exon 6 (c. 495G>A) was detected by the alignment of sequences. Computational prediction program did not predict the silent mutation.

**Conclusion:**

This study investigated a novel important sequence variant in the beta-crystalline protein that causes autosomal dominant congenital cataract (ADCC) in Pakistani families. Thus, our study enlarges the *CRYBB2* mutation spectrum and associated phenotypes to help clinical diagnosis of human genetic diseases.

## INTRODUCTION

Childhood congenital cataracts (CCC) refer to the lens opacity that appears at birth. A congenital cataract is among the more preventable causes of blindness and impaired vision in childhood around the world^1^. It is accountable for 14% of all childhood occurrences of blindness worldwide.^2^ Childhood Congenital cataracts represent around 22.3% of congenital cataracts worldwide, while the incidence of pediatric cataracts among the children attending eye outpatient clinics in Pakistan is 11.69%.^3^,^4^

Cataracts are the major cause of bilateral blindness (VA<3/60) in 46.5% of all instances. Shaikh S.P. et al. (2005) examined the pattern of eye diseases and prevalence in children aged five to fifteen. They investigated 5110 individuals in the Bazzertaline Area, South Karachi, Pakistan 2003. They noted a 0.27% prevalence of bilateral blindness, with cataracts as the major contributing factor and 2.2% prevalence of low vision and uncorrected refractive error as the main contributor. Additionally, they found 1.72% had a higher rate of visual impairment in girls than in boys.^5^ A study was also conducted by Naz et al. on the prevalence at Layton Rahmatullah Benevolent Trust Free Eye Hospital (LRBT). From October 2013 to April 2014, Lahore, Pakistan, examined 38,000 children under 15 years and found cataracts in 120 (0.3%) cases.^6^

Congenital cataract was managed using the corneal approach 20G vitrectomy system, with no surgical complications. No iris prolapsed, the anterior chamber was intact, and the incision fit. On the first postoperative day, four eyes showed conjunctival obstruction at the incision site and eight eyes showed a minor anterior chamber response. These symptoms disappeared one week later. Throughout the entire follow-up period, no other postoperative abnormalities, such as an inflammatory layer in the anterior chamber (AC), an asymmetrical pupil, posterior or anterior syneache, or visual axis opacification were observed follow-up period, no other postoperative abnormalities were observed, such as an inflammatory layer in the anterior chamber (AC), an asymmetrical pupil, posterior or anterior syneache or visual axis opacification. This is done to reduce the annoyance and risk of General Anesthesia to the children and their parents.^7^

Childhood congenital cataract (CCC) has been linked to over above100 genes and 200 loci. Childhood congenital cataracts are largely considered highly genotypically and phenotypically variable due to the significant number of loci and genes involved. Patients with the same mutations may have varied lens morphology, and different mutations may cause comparable characteristics.^8^ Genetic factors, low birth weight babies, viral infection during pregnancy, metabolic disorders, abnormalities of chromosomes, and hypoxia are prominent risk factors for congenital cataracts. Autosomal dominant (AD) inheritance is more common, whereas X-linked & autosomal recessive (AR) inheritance was also found.^9^

Autosomal dominant congenital cataracts (ADCC) seem to be the more prevalent kind of congenital cataracts, accounting for half of most cases. For several basic types of ADCC, at least 42 loci have been reported. Shiels & Hejtmancik evaluated nine, while the others were reported more subsequently.^10^,^11^ Although some of these forms have different characteristics, there is a lot of phenotypic overlap.^12^

To our understanding, CC formation may result from mutations in the gap junction, cytoskeletal, transcription factor, or crystallin genes. Amblyopia to sensory loss can result from severe CC. Surgery involves highly skilled professionals and has inherent hazards and potential problems, particularly in young patients. Another contentious issue is the timing of operative therapy, which results in significant yearly costs and a poor prognosis. However, the one effective CC therapy was the surgical removal of cataracts. Therefore, more research into the proteins in CC that cause mutations should result in a better understanding of the processes involved in cataractogenesis and novel therapeutic approaches to prevent lens opacification.^13^

Crystalline are the main constitutive lens proteins accounting for 90% of the eye lenses that help to retain lens transparency. α-crystalline, which function as chaperones, and the βγ-crystalline which has high stability of β-sheets belong to two crystallin families. β-crystallin family encoded the *CRYBB1, CRYBA1, CRYBB2*, and *CRYBA4* genes. β-crystalline mutations cause congenital cataracts, increasing proteolysis and vulnerability to stress, decreasing protein solubility, altering the protein-protein interface network, or changing the oligomeric equilibrium and causing lens opacity.^14^

Thus, this study shows the novel silent mutation (c. 495G>A/ p. G161G) in exon 6 of the *CRYBB2*gene. This mutation was predicted in Pakistani family members with Autosomal dominant congenital cataracts (ADCC). None of the previously reported mutations related to ADCC were found in any members of the families. Our study assists with the known mutation of *CRYBB2*-6 related to ADCC.

## METHODS

Two Pakistani families were included comprising eleven affected members. These families were recruited from the LRBT (Layton Rahmatullah Benevolent Trust) Ophthalmic Hospital, Mansehra, Pakistan.

### Ethical Approval:

This study, which was conducted under the terms of the 1975 Helsinki Declaration and updated in 2013, has been approved by the Institutional Bioethical Committee of the LRBT (Layton Rahmatullah Benevolent Trust) Ophthalmic Hospital in Mansehra, Pakistan (Ref No.: Dir A&R/Notifications/HU/2019/20).^15^ The consent was given directly by each person or their guardians, parents, or legal representatives. Complete descriptions by birth complications or clinical features of individuals were recorded. All subjects who were available underwent a complete ocular examination. Fundus and Slit-lamp examination, best-corrected visual acuity assessment, retinoscopy, and anterior segment biometry measures with an intraocular lens were included.

### Extraction of genomic DNA from blood samples:

Venous blood of (300 μL) from each participant of both families were taken in the tube of EDTA. DNA was isolated from the whole genome manually through phenol-chloroform method.^16^ Further, the quality and quantity of genomic DNA were confirmed with the 1% (TAE) electrophoresis method. DNA extracted was kept at −20°C till further process.

### PCR Amplification:

A sample of DNA from each affected family’s members were amplified by PCR, and the selected gene was sequenced as (C*RYBB2*_ex6 NM_000496). Details of primer and conditions of PCR for the *CRYBB2-*6gene are described somewhere. PCR primers that amplify regions surrounding probable loci have been prepared using Invitrogen Kit. Table-I shows the primer that was used to amplify *CRYBB2* by PCR. *CRYBB2* exon 6 with (NM_000496) was used following PCR amplification of genomic DNA in 25μL reaction volumes at 94°C for 5 minutes and 35 cycles of 94°C for 1 minute, 55°C for 45 seconds or one minute and 72°C for two minutes followed by an extension of 72°C for ten minutes.

**Table-I:** PCR Amplification of the Human CRYBB2 gene using oligonucleotide as a Primer.

Description of Gene	Sequence of Primer (5’-3’)	Size of amplified fragment
*CRYBB2*-6-L	CCCCTCGTTCACCCTCCCTCA	400bp
*CRYBB2*-6-R	CACTGTGTCCAAGGTCACACAGCTAAGC	

### Variation Validation and Sanger sequencing of the individuals:

**Fig.1:**
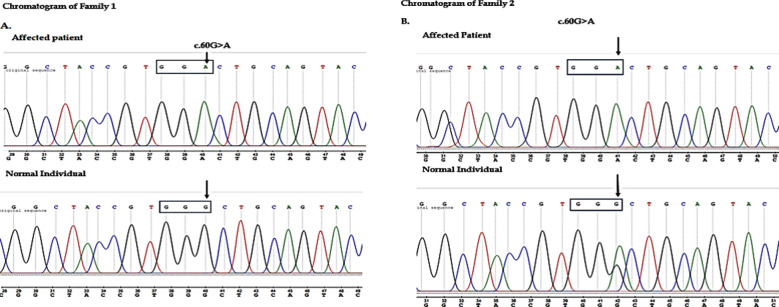
Family 1 and 2 sequencing result.

The amplified PCR products of affected and unaffected family members were successfully sequenced using the Sanger sequencing method. DNA was detected using the Applied Biosystems ABI 3730XL DNA Genetic Analyzer.^17^ Sequencing results of all samples of both families were evaluated to confirm the candidate variants by aligning the nucleotide sequence of all the samples of enlisted patients and normal members of the family members to a known reference sequence in the NCBI database. The nucleotide and reference sequence alignment showed the silent mutation in the *CRYBB2*-6 gene (Fig.1). A genomic website that searches and displays genomic variations from was used dbSNP, dbVar , and ClinVar datasets. In searches, the gene’s symbol, chromosome location, phenotype, or variant IDs from dbSNP and dbVar can all be utilized. The browser allows you to examine the findings in a dynamic graphical sequence viewer, including descriptive variant tables.

### Pathogenicity Evaluation of CCC Variants:

Multiple bioinformatics techniques were used to filter the data of candidate variants. The variant frequency was estimated from the databases of 1,000 Genomes, gnomAD, and dbSNP. The variant frequency was estimated from 1,000 Genomes, gnomAD, dbSNP and ClinVar databases. All alleles with a minor frequency of an allele (MAF) of over 0.0002 have been eliminated. The frequency was calculated using the CCs incidence rate (2.2/10,000).^18^

The prediction of the Splice Site program estimated the pathogenicity of the mutation in synonymous variant in exon. The refined results were checked against the Cat-Map database and the Human Gene Mutation Database to see if the candidate variants had discovered previously been reported.^19^ Thus, the candidate variant was not reported in the literature or database. Furthermore, It was using the SWISS-MODEL tool and a model for determining structures of No. AF-Q9H444-F1 and 1blb, a homologous *CRYBB2*-mutant (Gly)and wild-type (Gly) of the βB2-crystalline protein structures were generated (Fig 5).^20^ By using such databases the *CRYBB2* c.495G>A shows a significant and non-pathogenic mutation and specified the phenotypes of the patients and families hereditary fashion.

**Fig.2:**
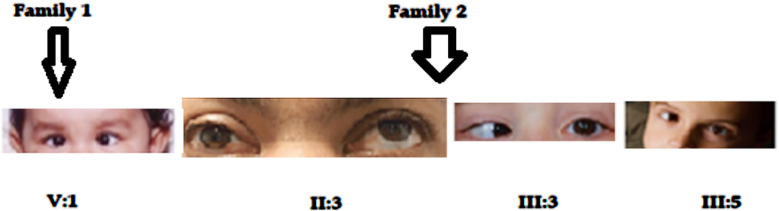
Image of patients showing nystagmus (V:1) and strabismus eyes (II:3); (III:3) & (III:5).

**Fig.3:**
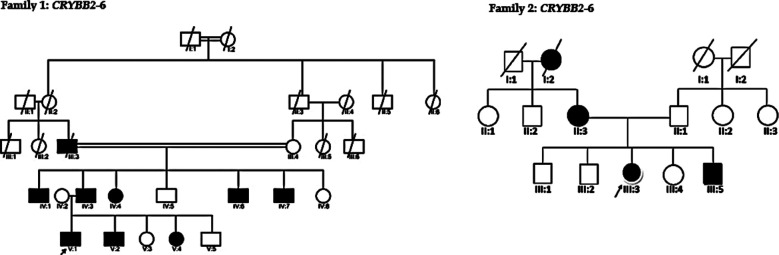
Autosomal Dominant Pedigrees of family 1 & 2.

**Fig.4:**
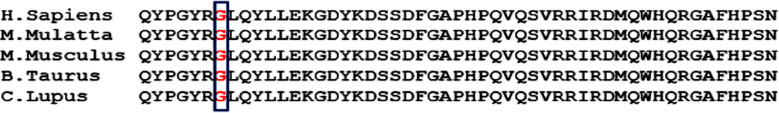
Protein alignment has shown conservation of p.161 amino acid position among different species. The p. 161 position is highlighted.

**Fig.5:**
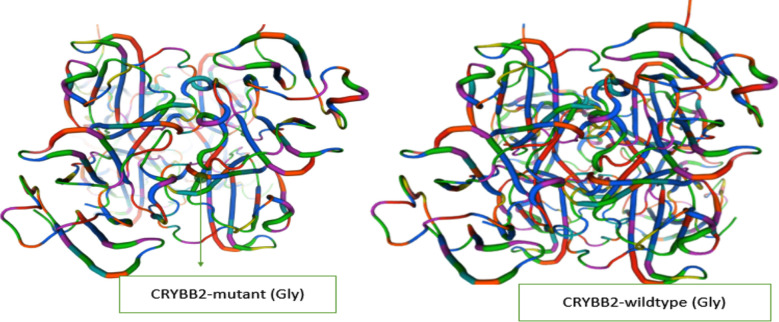
Swiss Model structure of mutant and normal βB2-crystalline.

## RESULTS

Eleven patients were analyzed (three females and seven males) with autosomal dominant congenital cataracts (ADCC) from two Pakistani families. The phenotype of lamellar cataracts was detected in a five-generation pedigree in family one. The proband of family one (V:1) was a two-year-old girl at admission time and was checked using a slit lighter red reflex examination by a pediatric eye specialist. Lamellar cataracts were found in all the affected members throughout the last three generations showing nystagmus eyes at birth. There have been no other visual or systemic anomalies (Fig.2). Similarly, the second family members, such as II:3, III:3, and III:5 were also diagnosed by a pediatric eye specialist with red reflex or slit light examination. All family members’ phenotypes were nuclear cataracts. Proband III:3 was embryonic nuclear cataracts. The affected members in family two showed strabismus eyes since birth and presented no other systemic alignment (Fig.2). The patient’s general characteristics and ophthalmic phenotypes, diagnostic tests and types of the identified affected members are as follows in Table-II.

**Table-II:** Clinical features of the patients of two Pakistani families.

# Features	IV:1	IV:3	IV:4	IV:6	IV:7	V:1	V:2	V:4	II:3	III:3	III:5
Age and sex	32Y/U	38/F	35Y/A	45Y/U	42Y/U	2Y/D	7Y/S	5Y/D	30Y/M	1Y/D	4Y/S
Incidence age	Since Birth	Since Birth	Since Birth	Since Birth	Since Birth	Since birth	Since Birth	Since Birth	Since Birth	Since birth	Since Birth
Microcornea	+	-	+	-	+	-	+	-	-	-	-
Strabismus	-	-	-	-	-	-	-	-	+	+	+
Nystagmus	-	+	-	+	-	+	-	+	-	-	-
Myopia	+	+	+	+	+	+	+	+	+	+	+
Red Reflex	-	+	-	+	+	-	-	-	+	-	-
Slit Lamp	+	-	+	-	-	+	+	+	-	+	+
Nuclear Cataract	-	-	-	-	-	-	-	-	+	+	+
Lamellar Cataract	+	+	+	+	+	+	+	+	-	-	-

+; Present, -; Absent.

### Pedigree Analysis:

Fig.3 shows family one and two pedigrees of three to five generations with childhood congenital cataracts. The pedigrees are transmitted in an autosomal dominant pattern. The family one (V:1) and family two (III:3) an arrow denotes probands. Males and females are represented by squares and circles, accordingly. Black and white icons, etc represent affected, and unaffected participants represent affected and unaffected participants. In family one, the cataract affected nine individuals who showed consanguinity in the second generation, while family one showed a non-consanguineous marriage and represented a single line among partners.

### Molecular Findings/Mutational Analysis:

The heterozygous mutation c.495TG>A (p. G161G) cosegregated with the phenotype, according to Sanger sequencing research. At this point, Sanger sequencing failed to detect the mutation in the 1,000 normal controls. Furthermore, we tested the mutation in the freshly released ExAC database of 63,000 controls exomes (http://exac.broadinstitute.org/) and found no mutations in this *CRYBB2* gene location. Our findings and clinical evidence suggested that the heterozygous mutation c 499TG>A (p. G161G) in exon 6 of the *CRYBB2* gene is the cause of congenital cataracts in these families. The amino acid involved in B2-crystallin is largely conserved across species, based on GenBank accession number NM_000496, the unique silent mutation c.495A in *CRYBB2* gene and discovered in our work (Fig.4). As a result, the silent mutation had no effect on the functioning of the *CRYBB2* protein.

Moreover, dbSNPdatabase, 1,000 Genomes database, and the GenomAD database did not report this reported silent mutation (Table-III). The pedigrees, families, co-segregation analyses, and sequence locations are presented in (Fig.3). In both families, the silent mutation *CRYBB2*: p. G161G appeared de novo. A basic glycine residue is replaced by the identical amino acid residue glycine (GGG→GGA). In exon 6 of the *CRYBB2* gene, the nucleotide G to A (G/A) polymorphism was reported. However, the coded amino acid remains unchanged.

**Table-III:** Mutation of *CRYBB2*-6 gene detection in two family members of CCC.

Family ID	Gene	Change in Exon-Base	Change in Amino Acid	Inheritance Type	dbSNP/1000G/GenomAD	Reported previously
IV:1	*CRYBB2*-6	c.495G>A	p.G161G	AD	NA/NA/NA	NA
IV:3	*CRYBB2*-6	c.495G>A	p.G161G	AD	NA/NA/NA	NA
IV:4	*CRYBB2*-6	c.495G>A	p.G161G	AD	NA/NA/NA	NA
IV:6	*CRYBB2*-6	c.495G>A	p.G161G	AD	NA/NA/NA	NA
IV:7	*CRYBB2*-6	c.495G>A	p.G161G	AD	NA/NA/NA	NA
V:1	*CRYBB2*-6	c.495G>A	p.G161G	AD	NA/NA/NA	NA
V:2	*CRYBB2*-6	c.495G>A	p.G161G	AD	NA/NA/NA	NA
V:4	*CRYBB2*-6	c.495G>A	p.G161G	AD	NA/NA/NA	NA
II:3	*CRYBB2*-6	c.495G>A	p.G161G	AD	NA/NA/NA	NA
III:3	*CRYBB2*-6	c.495G>A	p.G161G	AD	NA/NA/NA	NA
III:5	*CRYBB2*-6	c.495G>A	p.G161G	AD	NA/NA/NA	NA

NA: Not investigated; AD: Autosomal Dominant.

## DISCUSSION

Congenital cataract, distinguished by clinical and genetic variability, is primarily inherited by autosomal dominant inheritance.^21^ One-third of congenital cataract incidences are caused by genetic factors. 22With the advancement of molecular genetic tools in recent years, an increasing number of research organizations have focused on congenital cataract genetic abnormalities. Using direct Sanger sequencing, we discovered a new variant c.499GA (p.G161G) in the exon 6 of *CRYBB2* in a five generation Pakistani family with autosomal dominant congenital cataract.^22^

A Novel silent mutation was identified in *CRYBB2-*6 gene c.495G>A in all affected family members. Sanger sequencing showed a silent mutation in the C*RYBB2*gene of exon 6, which causes glycine to glycine change at the position of protein 161 (p. G161G). Moreover, this is a conserved region mutation that was not detected in GenomAD database, ClinVar, or 1,000 Genome project databases hence declaring it a novel mutation. Bioinformatic analysis showed that this mutation does not influence the structure and function of the protein. Thus, the result of *CRYBB2* exon 6 revealed that both codons encode for the same amino acid, glycine (GGG →GGA). This change leads to a novel silent mutation. A single base deletion was also found in both families affected members. The Sanger sequencing confirmed the silent mutation in all the infected individuals while not detected in healthy family members.

The *CRYBB2* gene may detect a variety of modifications. Recently, many genes have been responsible for causing congenital cataracts. But in this study, we have collected autosomal dominant congenital cataract families. Sanger sequencing detected gene mutation, which was used to relate all family members. We have reported a novel G→A silent mutation in the*CRYBB2*6^th^ exon related to both families’ phenotypes. These cataract phenotypes were previously reported to be associated with the *CRYBB2* gene, representing that the same gene mutation may cause different cataracts. To date, congenital cataracts have been linked to 15 mutations in the *CRYBB2* gene.^23^ Despite the same mutation, the cataract phenotypes for every family were quite diverse. S31W induced coronary cataracts in autosomal dominant congenital cataract patients.^24^ Q155X resulted in a wide range of phenotypes comprising cerulean, 25 polymorphic, Coppock-like, and sutural cataracts.^26^ But in this study, the morphology of one family is lamellar cataracts, which is different from many types of cataracts. Furthermore, in the 2^nd^ family, the phenotype we observed is similar to an Indian family with a distinct gene mutation and a central nuclear cataract.^27^ G161G is the first silent novel mutation reported for lamellar and nuclear cataracts, and thus, our Pakistani families provided new phenotypes related to *CRYBB2*. These findings and clinical evidence suggested that the heterozygous mutation c.499GA (p.G161G) in exon 6 of the *CRYBB2* gene is the cause of congenital cataracts in these families. In summary, the *CRYBB2* gene is important in forming the human lens and may be an important region susceptible to mutations that cause lens opacity.

More than 90% of the soluble proteins in the human eye lens are crystalline, with a percentage of 50%, 40%, and 30% for α-, β-, and γ- crystallins, correspondingly.^28^ The primary β-crystallin constituent, βB2-crystallin, is classified as a component of the γ/β/- superfamily. There are four Greek key patterns in both β-crystallin and γ--crystallin. A code in β-crystalline represents each Greek key pattern. A code in β-crystalline represents each Greek key pattern and consists of distinct exons. The *CRYBB2* consists of 6 exons: the 1^st^ one is untranslated, the 2^nd^ exon code for NH2-end addition, and the next 4 exons code for 1 Greek key pattern. The βB2-crystallin forms a homodimer and has 16 β loops in total, 8 in the NH2-end domain and 8 in the COOH-end domain, and they have several intermolecular interactions. Any mutation changing this intermolecular interaction will decrease *CRYBB2’*s solubility and stability, causing local binding to be lost, dimerization to be disrupted, and the ability to bind to other proteins to be impaired.^24^

Pakistani families associated with a congenital cataract of the nuclear and lamellar type, a unique silent mutation of the *CRYBB2* gene was found and described. Our understanding of the process behind cataract formation will be strengthened by more research on this cataract-related genetic abnormality and the variables that affect their various phenotypes, which will also shed light on the developmental biochemistry and biology of the lens. As stated, identical mutations in various families or the same mutation in the same family can cause cataracts with completely distinct morphologies and severity. Mutations in completely different genes may induce cataracts with the same clinical symptoms. Inherited congenital cataracts have an unknown genotype-phenotype relationship. This suggested that environmental factors or other genes could influence the appearance of cataract-causing mutation. Further research on genetic defects linked to cataracts as well as the elements that influence phenotypic variability and help us to understand better how cataracts form and shed light on the lens’ embryonic biology and biochemistry.^29^

### Limitations of the study:

The reported mutations in the current study are based on the data from patients of two families belonging to District Mansehra KP, Pakistan. There is a need to verify the reported mutations to verify it in the study area. Hence some other mutations may also be found leading to CCC. A small sample size may affect the result.

## CONCLUSION

We can conclude that congenital cataract families from Pakistan have a specific silent mutation. These findings point out the unique role of the *CRYBB2* protein and widen the spectrum of disease-related mutations in the region. The *CRYB*B2 gene’s function in congenital cataracts may be better understood as a result of the finding of a mutation in the gene. The research’s findings could also show a phenotype-genotype relationship and support the development of more accurate and precise treatments and diagnoses, carrier screening for the horizontal and vertical transfer of congenital cataracts, and appropriate genetic counseling methods. The goal of future study might be to recognize congenital cataract families which could produce similar variants to compare their differences & similarities.

### Authors’ Contribution:

**MH** designed the study, conducted experiments, collected the data, and wrote the article’s first draft. **MH** is responsible and accountable for the accuracy and integrity of the work. **KM, MK** conceived the idea, supervised the study, and reviewed and edited the final draft. **AZ** software and formal analysis reviewed and approved final draft. All the authors read, reviewed, & approved the final manuscript for publication.
